# Burnei’s technique of femoral neck variation and valgisation by using the intramedullary rod in Osteogenesis imperfecta

**Published:** 2014

**Authors:** I Georgescu, Șt Gavriliu, I Nepaliuc, L Munteanu, I Țiripa, R Ghiță, E Japie, S Hamei, C Dughilă, M Macadon

**Affiliations:** * ”M. S. Curie” Children’s Clinical Emergency Hospital, Bucharest, Romania; ** ”Nicolae Testemiţeanu” State University of Medicine and Pharmacy, Chişinău, Moldova Rep.; *** Children’s Clinical Hospital, Brașov, Romania

**Keywords:** Osteogenesis imperfecta, coxa vara, coxa valga, only rod correction, Burnei’s technique

## Abstract

**Background:** Varus or valgus deviations of the femoral neck in osteogenesis imperfecta have been an ignored chapter because the classic correction procedures were applied in medical practice with unsatisfying results. Until the use of telescopic rods, coronal deviations remained unsolved and the distal configuration of the proximal femoral extremity remained uncorrected or partially corrected, which required an extensive use of the wheel chair or bed immobilization of the patient. The concomitant correction of the complex deformities, coxa vara/valga and femoral integrated configuration, have been a progress which allowed the patients to walk with or without support.

**Purpose:** The purpose of this study is to present the Burnei’s technique, a therapeutic alternative in deformity corrections of the varus or valgus hip in children with osteogenesis imperfecta.

**Study design:** The paper is about a retrospective study done in a single center, which analyses Burnei technique and other procedures described in literature.

**Patient sample:** The content of the article is based on a 12 years experience on a batch of 51 patients with osteogenesis imperfecta from which 10 patients (13 hips) presented frontal plane deviations of the femoral neck.

**Outcome measures:** All the patients with osteogenesis imperfecta who presented coxa vara or valga were submitted to investigations with the purpose of measuring blood loss, the possibility of extending the surgical intervention to the leg, the association of severe deformities of the proximal extremity of the femur and the necessity of postoperative intensive care.

**Burnei’s technique:** The operation was first performed in 2002. A subtrochanteric osteotomy was made in an oblique cut, from the internal side to the external side and from proximal to distal for coxa vara, or by using a cuneiform resection associated with muscular disinsertions. Only telescopic rods were used for osteosynthesis.

**Discussions:** There are a few articles in literature, which approach corrections of vara or valgus deviations in osteogenesis imperfecta. Some of them are the techniques described by Finidori, Wagner and Fassier.

**Conclusions:** Burnei’s technique is simple; it corrects the varus and valgus deviations concomitantly with Sofield-Millar. Even though only a telescopic rod is used, no stress fractures were seen postoperatively, deviation recurrence or assembly loss.

## Introduction

Proximal femoral deformities in children with osteogenesis imperfecta (OI) vary depending on the clinical form of the disease. The less the bone resistance, the more the circle deformity-fracture-malunion is obvious and the coronal deviation, varus or valgus, are accompanied by severe distortions of the intertrochanteric and subtrochanteric regions as well as of the femoral neck. 21% of the patients with OI presented coronal deviations of the proximal femoral extremity. The type III form of OI was the most encountered form, followed by type IV and I. Varus deviation was more frequent (8.8%) than the varus one (6.8%). Severe distortions of the proximal femur were encountered in 5 patients (6 hips), all with type III OI. Once the femoral shaft configuration was corrected by using the Sheffield or Fassier-Duval rods, it is indicated that varus or valgus deformities, as well as all the distortions must be corrected.

Several techniques for the correction of these deformities were described in literature. Initially, classic techniques were used, consisting in intertrochanteric osteotomies and internal fixation by using plates and screws, nail-plates, pins etc., but all of them proved to be unsatisfying and they are no longer in use. In 1988, Finidori first described a procedure in which the coxa vara deformity was corrected by using a telescopic rod, which was inserted retrogradely through the cortex of the lateral side of the proximal femur being exteriorized at the base of the femoral neck. In 2003, Fassier combined the Finidori procedure with the Wagner synthesis, which consisted in fixing the osteotomy site with 2 pins passed through the femoral neck. The 2 pins were molded to the femoral shaft and fixed by using 2 cerclage wires. Fassier resected a triangular bony fragment from the distal femur to enhance stability. Recently, a system consisting in plate and screws has been conceived, associating a “gap-nail” intramedullary rod that enhances stability and avoids stress fractures and it is designed for pediatric use.

**Burnei’s technique**

**Fig. 1:**
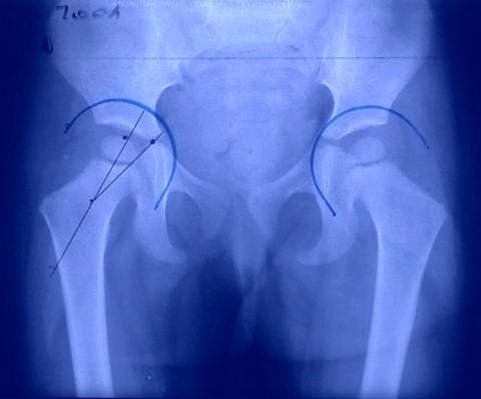
The head center-acetabulum center discrepancy (HC-ACD) appreciates the rate of the inclination angle diminution from 150° to 130° and it indicates the need of associating a muscle relaxation to the osteotomy. The normal value of HC-ACD is between +/- 15°. A value bigger than 15° represents the coxa vara and a value lower than -15° represents the coxa valga when the acetabulum is normal (after Gh. Burnei).

Since 2002, professor Burnei has been applying an original and simple technique, which allows the correction of both coxa vara and coxa valga. In 2008, an article with the title “Osteogenesis Imperfecta: Diagnosis and Treatment” was published in JAAOS, in which the scheme for coxa vara correction was communicated [**1**]. This technique keeps in mind the degree of the deformity, the femoral neck-femoral shaft angle and, depending on its value, the angle on which the intamedullary rod is inserted can be determined so that the femoral neck forms an angle of 130° with the femoral shaft, and the head center-acetabulum center discrepancy (HC-ACD) is of less than 20° (**Fig. 1**).

**Fig. 2:**
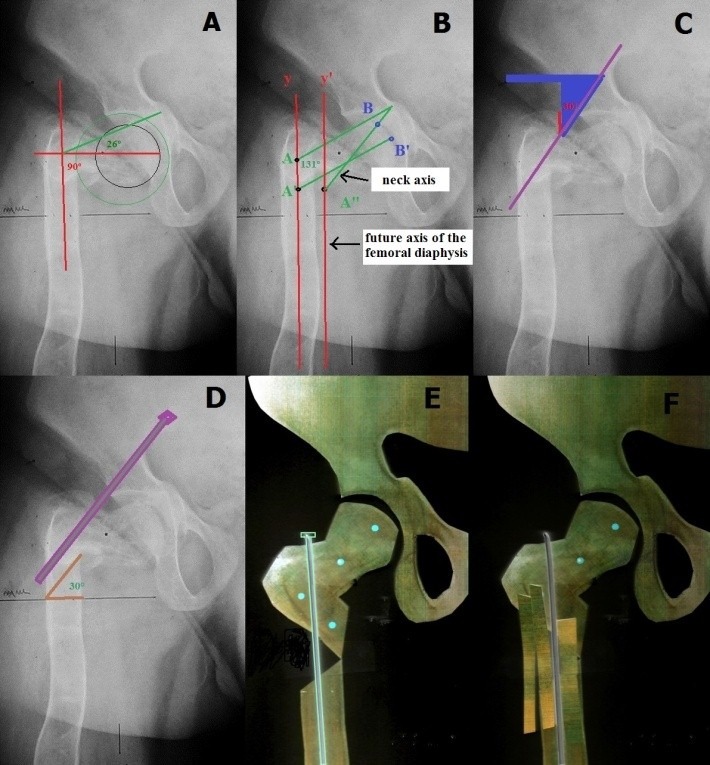
**A** The inclination angle of the femoral head must be 90+26=116°, on which 10-15° are added: between 126 and 131°. The neck’s axis will be translated distally, the point of junction between the neck’s axis and the shaft’s axis descending to allow the normal positioning of the femoral head in the acetabulum. **B**. Point A descends and is medialized taking A’ position, and the femoral head is centered into the acetabulum: B takes B’. The shaft’s axis is medialized and y takes y’ position. **C** and **D**. The osteotomy is made 1-2 cm distally from the point the rod is inserted into the bone cortex, parallel with the rod’s trajectory, at an angle of 30°. **E** and **F**. The telescopic rod inserted through the piriformis fossa, exteriorized through the lateral cortex of the proximal femur follows the intramedullary trajectory into the distal fragment of the femur. The uncovered portion of the rod is grafted

Coxa vara was diagnosed at an inclination angle of <120° and the coxa valga when the inclination angle was >150°. Preoperatively, the degree of correction was established as it follows:

A. **FOR VARUS** (**Fig. 2**)

**Fig. 3:**
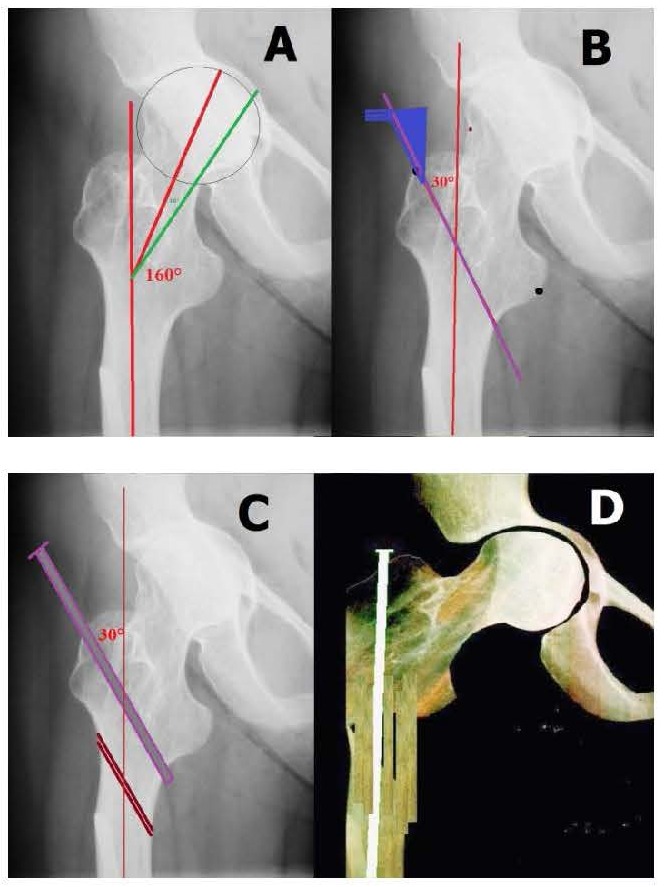
**A** and **B** HC-ACD is of 16°. At an angle of 130°, correlated with allowed HC-ACD values, in this case +15°, the femoral neck can be positioned at 129°. **C** and **D**. The telescopic rod inserted through the tip of the greater trochanter, exteriorized through the medial cortex of the proximal femur, follows the intramedullary trajectory into the distal fragment of the femur. The uncovered portion of the rod is grafted. The greater trochanter is lateralized, the neck is descended and the femoral head’s center which was at 130° will correspond to a normal position

B. **FOR VALGUS** (**Fig. 3**)

The procedure used the Watson-Jones approach: the oblique portion of the incision was placed in a more posterior place.

1. *The technique for coxa vara*

**Fig. 4:**
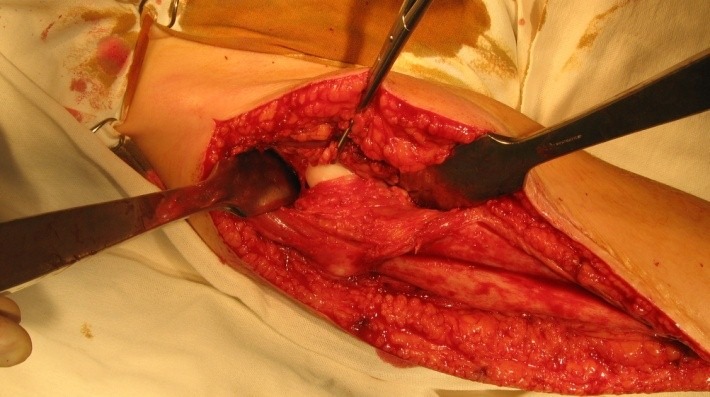
After the elevators were distally and proximally placed to the articular capsule, the capsulotomy was done cranially and in a hemicircumferential manner

The virtual space between the pelvic trochanteric muscles and the articular capsule was dissociated (**Fig. 4**) and an elevator was inserted.

**Fig. 5:**
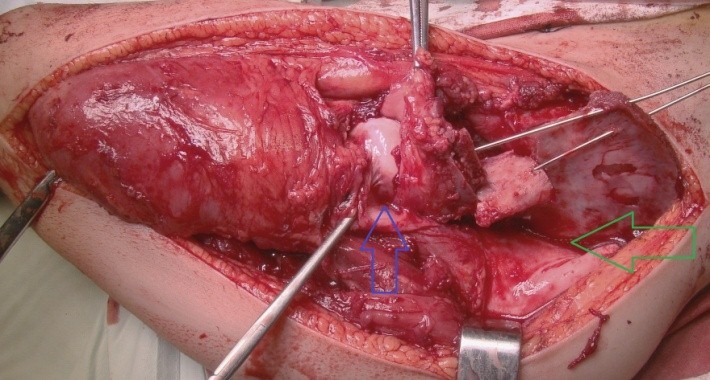
After the osteotomy was done at 130°, the HC-ACD was in normal range, but the acetabulum proved to be insufficient, which led to the necessity of a triple osteotomy of the pelvis with acetabuloplasty and autogenous bone graft fixed with 2 K pins. The periosteal disinsertion and the cranial hemicircumferential capsulotomy allowed the restoration of the hip’s normal configuration, which reduced the risk of complications and no other fixation methods were needed beside the telescopic rod

The Sartorius muscle was detached from the ventro-cranial iliac spine, sectioning a portion from the bone by using a power blade and the Rectus femoris muscle was detached from the ventro-caudal iliac spine. The space between the capsule and the psoas muscle was penetrated. The periosteum was sectioned off the external side of the coxal bone through an incision under the iliac crest and a deperiostation by disinsertion was done. The articular capsule was sectioned transversally, hemicircumferential on the cranial half (**Fig. 5**).

**Fig. 6:**
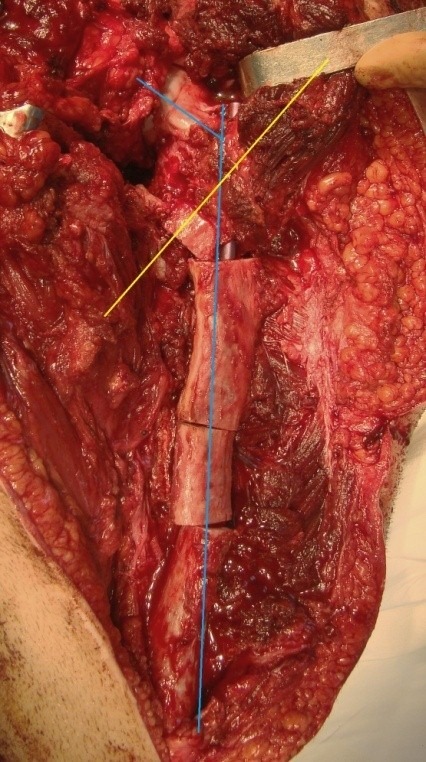
Intraoperative aspect after the oblique osteotomy for the correction of varus deviation

The osteotomy was subtrochanteric, obliquely, upward from lateral to medial performed, at the calculated angle for correction. The rod was obliquely inserted, anterograde through the piriformis fossa and it was exteriorized through the external cortex of the proximal femur while regarding the correction angle (**Fig. 6**). The Sheffield or Fassier-Duval telescopic rod was inserted through the medullary canal of the shaft by using a classic approach or a minimally invasive one.

The deperiostation by disinsertion of the pelvic trochanteric muscles and the cranial hemicircumferential incision of the articular capsule reduced the tension exerted on the fixation system and on the proximal extremity of the femur. The telescopic rod fixation without other implants was sufficient. The subtrochanteric oblique osteotomy allowed the proximal part of the section to be aligned to the internal cortex, the femoral shortening being under 1 cm.

2. *The technique for coxa valga*

A medial muscular relaxation was performed if the adductor muscles of the thigh were contracted and retracted: transversely approach, adductor fascia sectioning (gracilis, adductor longus and iliopsoas muscles). The caudal hemicircumferential sectioning of the articular capsule could be performed by using this approach or by using the Watson-Jones one, and it was indicated when the femoral neck’s positioning was at a 130° angle to the diaphysis hindered by the capsule’s retraction or by a dysplastic, insufficient acetabulum. Using a Watson-Jones approach, the greater trochanter’s tip was exposed.

The subtrochanteric osteotomy was performed in an oblique manner, downwards, from lateral to medial, vice-versa to coxa vara. The rod was inserted obliquely, under an angle calculated preoperatively, according to the required angle. The point of insertion was on the greater trochanter and the rod’s exteriorization was performed through the internal cortex, following the intramedullary femoral shaft’s trajectory. In this case, the proximal part of the section was aligned to the external cortex.

After the oblique osteotomies were performed, the uncovered portion of the rod was grafted by using autografts or allografts. If the oblique osteotomy could not be performed due to the thigh’s muscle tension, especially in coxa vara, a cuneiform resection from the distal femur was performed, with the base oriented externally for coxa vara and internally for coxa valga.

**Fig. 7:**
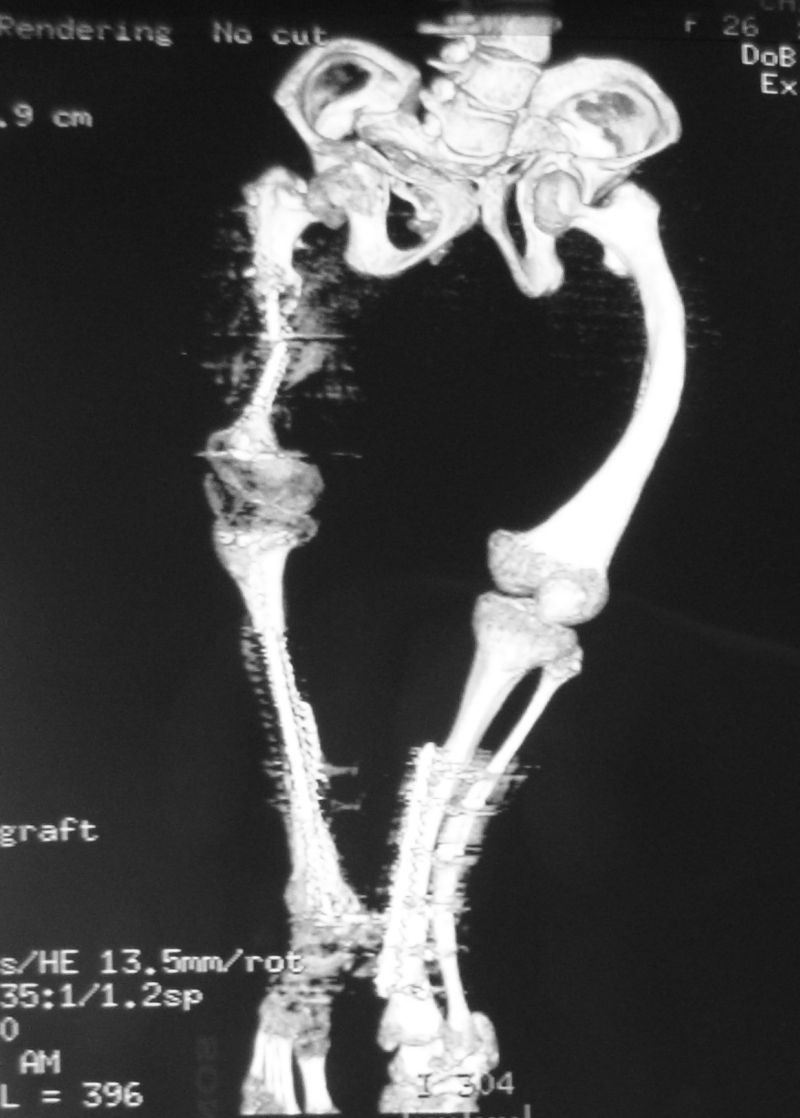
The 3D-CT showed a severe distortion of the proximal right femoral extremity with a hypoplastic configuration and an apparent shorter right femur

Intraoperatively, a surprise came that the inclination angles did not match the ones calculated preoperatively, due to severe distortions of the proximal femoral extremity, even though the preoperative evaluation included a 3D-CT (**Fig. 7**).

Giving this situation, the surgeon must calculate the correction angle intraoperatively. The severe distortions sometimes require associating inter- and subtrochanteric osteotomies so that in the proximal femur’s configuration, the inclination and anteversion angles are restored. If there is an acetabulum dysplasia, hip dislocation or hip subluxation is present, during the same operation, an ostearthroplastic hip reconstruction is performed to ensure an anatomical congruence of the femoral head-acetabulum.

## Discussions

Generalities

There are not many articles that approach the issue of proximal femoral extremity deformations in Osteogenesis Imperfecta, (coxa vara more frequent than coxa valga), which means the adaptation of the existing surgical technique to fragile bone, predisposed to fractures.

In 2006, Aarabi et al published the results of a study done on 283 patients with Osteogenesis Imperfecta, the main concern being coxa vara [**2**]. The authors concluded that these patients had a higher rate of coxa vara (10.2%), periodical clinical and radiologic reevaluations being mandatory, especially in patients with severe disease forms (type III), and in those in whom intramedullary rods were implanted in the femur [**2**]. The intramedullary rod’s presence in the femur, especially when it is incorrectly positioned, predisposes to the appearance of varus deformity of the femoral neck in relation with the diaphysis [**3**,**4**]. The unoperated patients also showed this predisposition due to bone fragility and microfractures present in the proximal femur, coxa vara having an auto-aggravating nature [**5**].

In order to discover the deformity, the angle between the femoral neck’s axis and the shaft’s axis was measured and if its value was less than 110, coxa vara was diagnosed [**2**,**5**,**6**]. A severe curvature of the proximal femoral diaphysis may be erroneously interpreted on an X-ray image as being coxa vara [**5**]. In order to determine the diagnosis, a lateral X-ray image of the pelvis can be performed [**7**].

Clinically, the abduction restriction of the affected hip could be observed, obviously, in walking patients who have the Trendelenburg sign. Coxa vara enhances the functional deficits due to bone fragility and the existing skeletal deformities in a patient with Osteogenesis Imperfecta, which leads to the necessity of a difficult surgical correction on osteoporotic bone.

The correction of coxa vara/valga on telescopic rod

In 1988, Finidori proposed a correction technique for coxa vara on telescopic rod based on subtrochanteric osteotomy on the proximal extremity of the femur, the rod being retrogradely passed through the lateral cortex and exteriorized at the base of the femoral neck.

In 1999, Fassier and Glorieux promoted Finidori’s technique for *coxa vara* >90°, and when the angle between the femoral neck and femoral diaphysis was of <90°, they proposed the classical correction and internal fixation with a pediatric nail-plate; this one had to be extracted immediately after the appearance of consolidation signs at the osteotomy site, and had to be replaced with a intramedullary rod, because of the risk of fracture at the inferior pole of the plate [**8**]. In reality, this last procedure of synthesis on osteopenic bone was extremely risky and had to be avoided [**5**].

In 2003, Fassier published an original procedure for coxa vara correction on patients with Osteogenesis Imperfecta, the procedure being the result of mixing two techniques:

1. Finidori’s technique, communicated in 1998, consisting in a subtrochanteric osteotomy and synthesis with a telescopic rod passed through the external cortex of the proximal femoral extremity and exteriorized at the base of the femoral neck;

2. The synthesis method described by Wagner in 1978, consisting in the fixation of the osteotomy site with two K pins passed through the femoral neck.

The two pins were useful even for the manipulation of the proximal fragment of the femur after the osteotomy had been performed, so that the fragment was correctly positioned. Fassier added an artifice, which allows the resection of a triangular bone fragment from the distal femoral fragment, ensuring a better stability [**2**,**5**,**7**]. After obtaining the right position, the 2 pins were molded to the femoral shaft and fixed by using 2 cerclage wires. [**2**,**5**,**7**]. For intramedullary fixation, Fassier used both telescopic rods with T extremities and Fassier-Duval rods.

In 2008, Fassier et al published their results after applying the technique described by the first author for the correction of coxa vara in patients with osteopenia, 16 children being examined, respectively 18 hips with osteogenesis imperfecta and 3 hips with fibrous dysplasia, operated during 1996-2005 [**7**]. The rate of complications was 12%, being related with the implant. The conclusion of the study was that Fassier’s procedure ensured a satisfying correction for coxa vara in children with osteopenia [**7**].

A complex gap-nail implant based on Fassier’s technique was recently introduced on the medical market under the form of endo-exo medullary system, consisting in a plate with screws that made the common body with a paediatric intramedullary rod. This plate-rod system has some advantages, making this implant an alternative method of synthesis for patients with Osteogenesis Imperfecta and varus/valgus proximal femur deformities:

- It is suitable for small diaphysis;

- Lower risk of stress fractures occurrence;

- Greater implant stability in fragile bones.

In our Clinic’s experience, 11 out of 51 patients with osteogenesis imperfecta had deviations of the proximal femur in the coronal plan, 3 (3 hips) associating hip dysplasia. 2 (2 hips) were operated on and in the third one, an adult, the 3D-CT and MRI exams revealed the acetabulum aplasia and the disappearance of the articular cartilage and the patient was not operated on.

Using Burnei’s technique for the correction of the proximal femur deviations in the coronal plan, 5 patients (7 hips) had been operated for coxa vara and 5 patients (6 hips) for coxa valga, all of them suffering from Osteogenesis Imperfecta. The results were very good, without complications like stress fractures or fixation montage deterioration, due to the stability gained by the disinsertion of pelvic trochanteric muscles and capsulotomy. After only one intervention, a valgus hypercorrection from 170° to 110° had been recorded. This procedure has the advantage of being easy to associate with femur correction osteotomies, other additional implants besides the intramedullary rod not being required.

Starting with 2002 until present, the correction for coxa vara or coxa valga in patients with Osteogenesis Imperfecta has been simultaneously performed in our Clinic by using the Sofield-Millar technique and by implanting the telescopic rod as it follows:

1. Oblique subtrochanteric osteotomy or cuneiform resection;

2. Pelvic trochanteric muscles disinsertion-deperiostation and cranial hemicircumferential capsulotomy for coxa vara;

3. Medial muscle relaxation of the thigh consisting in adductor longus, gracilis and iliopsoas muscles tenotomies in case muscle contraction and retraction was presently associated with caudal hemicircumferential capsulotomy;

4. Autografts and allografts on the uncovered portion of the rod;

5. Osteotomy site fixation by using only a Sheffield telescopic rod, with no other implants;

6. When dealing with coxa vara, the telescopic rod was inserted through the piriformis fossa, exteriorized through the lateral cortex of the proximal femur, afterwards following its trajectory through the medullary canal into the distal fragment of the femur;

7. When dealing with coxa valga, the rod is inserted through the greater trochanter and exteriorized through the internal cortex.

## Conclusions

There are multiple surgical techniques aimed to correct coxa vara/valga. Burnei’s technique is easier to be done. It does not use other internal fixation materials besides the telescopic rod and avoids all complications that might arise from the tensions induced by the pelvic trochanteric muscles in coxa vara or by the adductor muscles in coxa valga. Diminishing the tension by using periosteal disinsertion of the pelvic trochanteric muscles and adductor muscles tenotomy avoids complications that might arise due to the tension produced by their contraction and retraction.

This paper was done inside The Sectorial Operational Program for Human Resources Development. (POSDRU) 2007-2013, EXCEL-FIN project, identification contract number: POSDRU/107/1.5/S/82839
